# A patient with Multiple myeloma and Renal cell carcinoma

**Published:** 2016-01-01

**Authors:** Farhad Shahi, Marziye Ghalamkari, Mehrzad Mirzania, Mahdi Khatuni

**Affiliations:** 1 Department of Hematology and Medical Oncology, Cancer Research Center, Cancer Institute, Imam Khomeini Hospital Complex, Tehran University of Medical Sciences, Tehran, Iran; 2 Internal Medicine Resident, Imam Khomeini Hospital Complex, Tehran University of Medical Sciences, Tehran, Iran

**Keywords:** Multiple myeloma, Renal cell carcinoma, Secondary hematologic malignancy

## Abstract

The coexistence of two malignancies is rarely seen. A little association between hematologic malignancies especially multiple myeloma and renal cell carcinoma has been reported in the recent past. Several case series revealed a bidirectional association between these two malignancies which may be due to the common risk factors, similar cytokine growth requirements and clinical presentation. Here, we aim to describe a patient who had multiple myeloma and in his work up renal cell carcinoma was found out incidentally. We would like to create awareness among clinicians for the coincidence of Renal cell carcinoma and Multiple myeloma.

## Introduction

 Multiple myeloma (MM) represents a malignant proliferation of monoclonal plasma cells that synthesize abnormal amounts of immunoglobulin or immunoglobulin fragments. Approximately, 24,050 new cases of myeloma were diagnosed in 2014 in the United States. Myeloma accounts for 1.3% of all malignancies in whites and 13% of all hematologic cancers.^1^

The median age at onset is approximately 65 years, although occasionally myeloma occurs as early as the second decade of life. The etiology of human myeloma is unknown. Environmental exposure to radiation and chemicals has been associated with an increased incidence of myeloma.^2^ Clinical manifestations are heterogeneous and include the formation of tumors, monoclonal immunoglobulin production, and decreased immunoglobulin secretion by normal plasma cells leading to hypogammaglobulinemia, impaired hematopoiesis resulting in anemia and other cytopenias, osteolytic bone disease, hypercalcemia and renal dysfunction. Renal cell carcinoma (RCC) is a result of malignant proliferation of the epithelial cells of proximal convoluted tubule of nephron and accounts for 95% of malignant neoplasm of kidney. The incidence is now nearly 65,000 cases annually in US.^3^

The development of secondary hematologic and solid malignancies such as acute leukemia, MDS, CLL, lymphoma and second solid tumor has been reported after therapy in MM patients. Also multiple primary malignancies like prostate, bladder and non Hodgkin lymphoma have been reported in patients with RCC.^4^ Cytogenetic abnormality, stem cell disorder, radiation therapy, chemotherapy and the malignancy itself may be prognostic risk factors for the development of secondary malignancy. The role of cytokines especially IL6 has been magnified in renal cell carcinoma cells which may stimulate myeloma cells and myeloma cells decrease after nephrectomy.^5^

There has been some case series about the relationship between RCC and MM. A cohort study revealed a bidirectional association between RCC and MM that lead to the same risk factors. These shared risk factors include: obesity, hypertension, and smoking. They share same lytic bone lesions and similar cytokine requirement.^6^

Here we discuss one patient with MM and RCC.

## CASE

**Figure 1:**
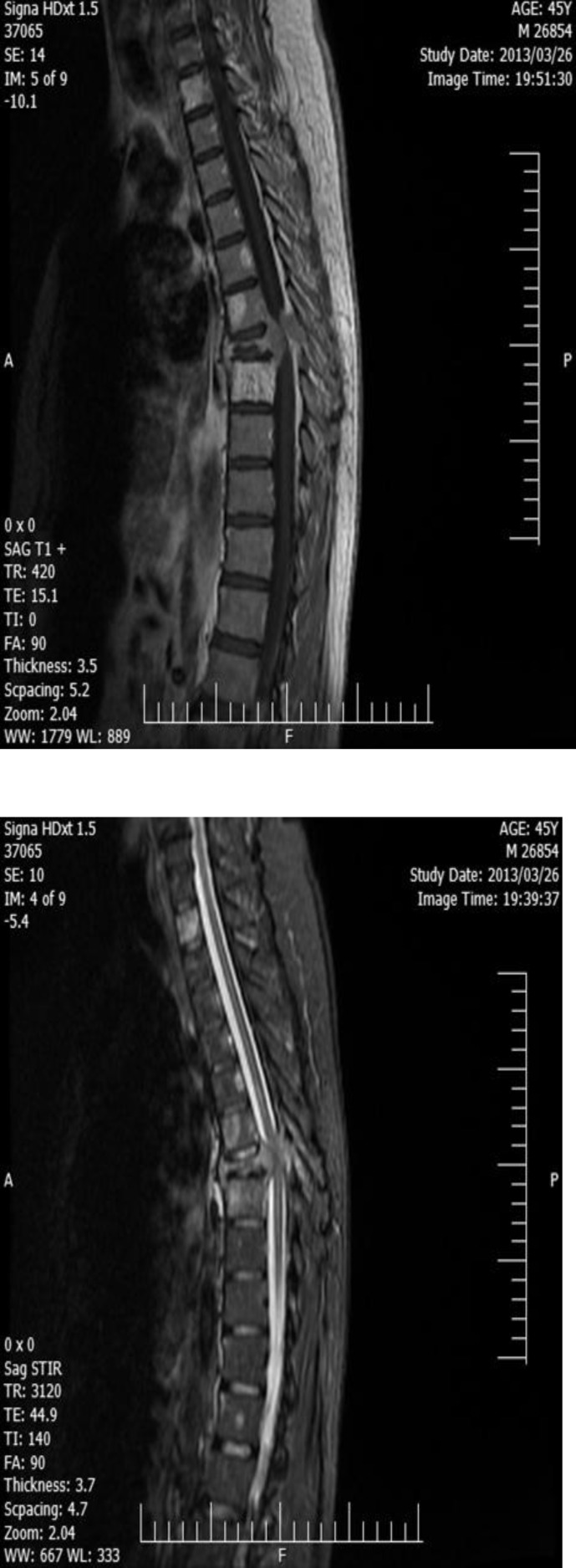
T7-spinal body destruction (pathological fracture)

 Our patient was a 46 year old man who initially presented with 3 month history of progressive back pain that eventually led to acute bilateral paraparesia. He was an office manager from north of Iran without any history of trauma or radiation and chemical exposure. He did not mention any specific medical or drug history. Significant findings on physical examination were point tenderness over lower thoracic area, paresthesia and paresis (muscle force: 3/5) of both lower extremities. Other examinations were normal. Radiological work up included: a thoracolumbar MRI which showed T7-spinal body destruction (pathological fracture) with compressive effect on spinal cord and multiple high intensity spinal bone lesions (Figure1).

He underwent emergent spinal surgery and fixation of the spine. The pathology of the resected T7-lesion showed “plasmacytoma”. The specimen was reevaluated by another pathologist and the diagnosis of plasmacytoma was confirmed.

**Figure 2:**
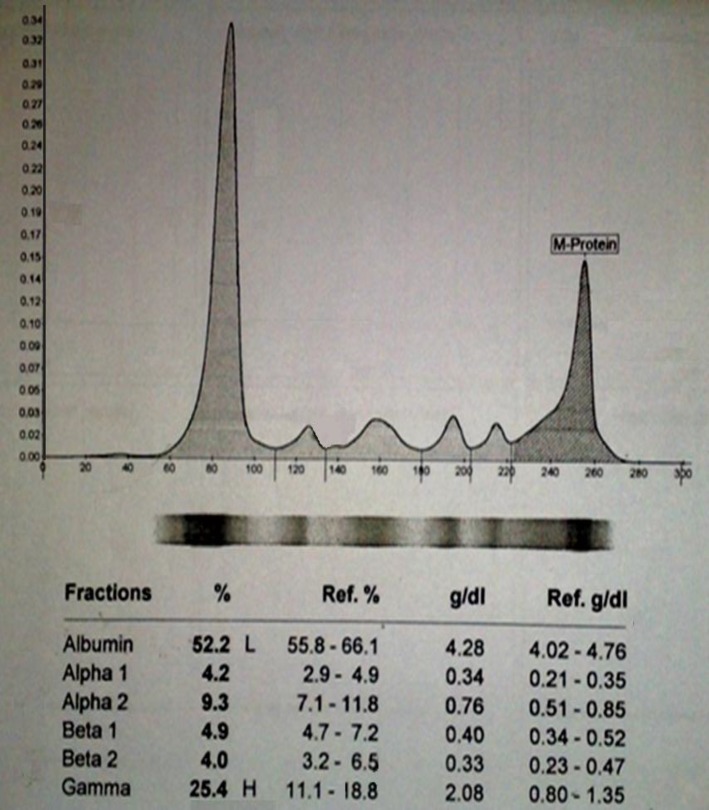
Serum protein electrophoresis revealed M spike

In further work up serum protein electrophoresis revealed M spike with IgG level 3764 mg/dl (700-1600) (Figure 2).

Urine protein electrophoresis showed elevated lambda light chain. Bone marrow aspiration revealed mild to moderate increased in plasma cells with atypical forms (12% moderate atypical plasma cells) confirming the diagnosis of multiple myeloma. Cytogenetic showed: 46XY, inv (9) (p11q12) compatible with apparently normal male. Other lab data was almost normal (Table 1).

Skull X-ray was normal without any punched out lesion. Spinal radiologic evaluations did not show any other abnormality except diffuse osteopenia. A Bone scan was negative.

During patient’s work up, an abdominal ultra-sonography revealed a mass in left kidney.

**Table 1:** Laboratory data at presentation of our patient

	**Our patient**	**Normal range**
**White blood cells (10** ^3^ **/μL)**	8	4.5-10
**Hemoglobin (gr/dl) **	13.2	13-15.5
**Creatinine (mg/dl)**	0.8	0.5 to 1.0
**Calcium (mg/dl)**	8.5	8.5-10.5
**ESR 1th hour (mm/h)**	35	<50
**LDH (U/L)**	369	50-150
**Albumin (g/dl)**	3.9	3.5-5.5
**B2-microglobulin (mg/l)**	1.7	<2

**Figure 3:**
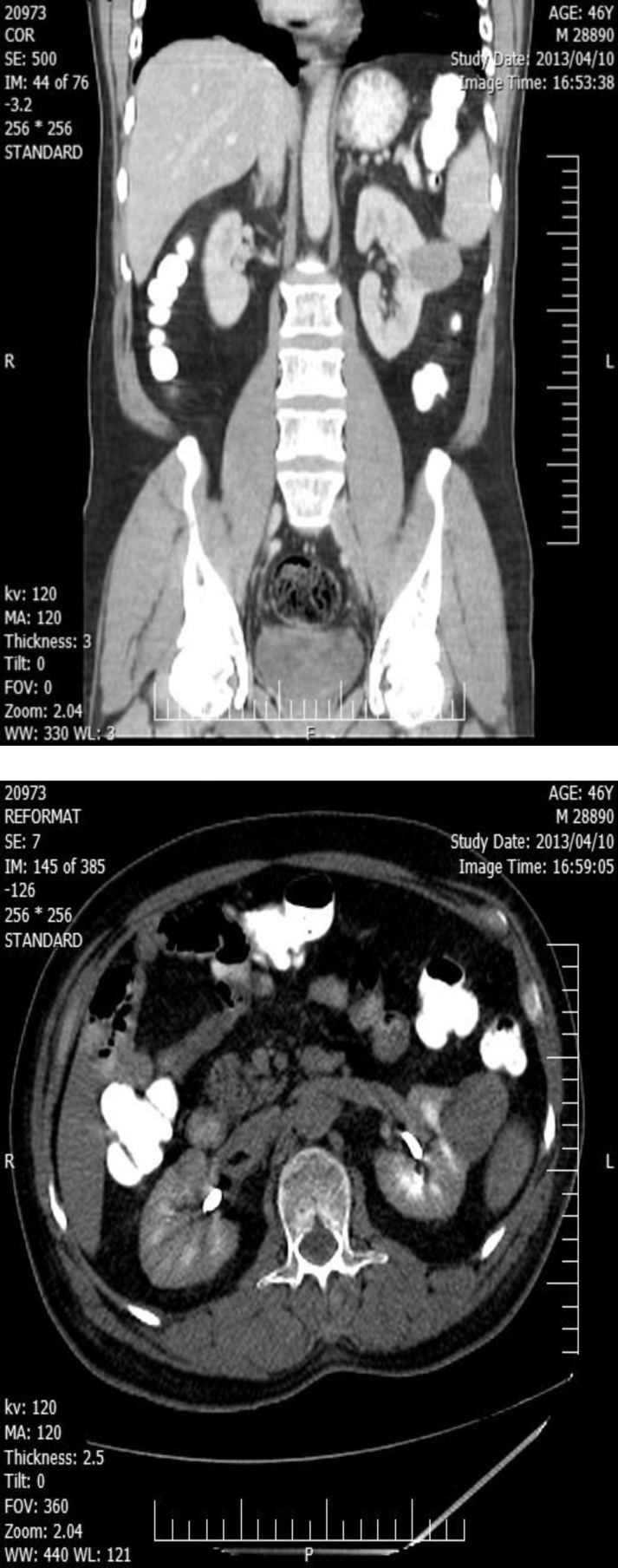
CT scan revealed a well-defined solid mass (34 x 46 x 36 mm) within the left kidney

CT scan showed a well-defined solid mass (34 x 46 x 36 mm) within the left kidney (Figure 3). Core needle biopsy of the mass was performed and pathology was consistent with RCC (papillary type).

The patient received radiation to spinal lesions and underwent partial left nephrectomy. The pathology showed a 5 cm renal cell carcinoma (Papillary type, nuclear grade 2) mass involving renal parenchyma without invasion to vascular or lymphatic system. He recovered well post operatively and subsequently started on chemotherapy for multiple myeloma (Vincristine, Adriamycin, and Dexamethasone). After four courses of chemotherapy, serum protein electrophoresis got normal; we changed chemotherapy to thalidomide (200/d) for the next four months.

## Discussion

 The incidence of second malignancies is rising due to the successful treatment of primary malignancies and increase life expectancy. In multiple myeloma, the risk of a secondary myelodysplastic syndrome or acute leukemia is approximately 3% at 5 years and 9% at 10 years. Some authors have suggested that higher cumulative doses of melphalane is a risk factor for developing secondary malignancy^7^ but our patient did not receive any chemotherapy before.

Sehgal reported a rare case of metastatic prostatic cancer to bone marrow and MM as secondary malignancy. He suggested that probably bone marrow microenvironment play a crucial role in the development of myeloma.^8^ Sporadic case reports have revealed dual malignancies occurring in renal cell carcinoma; such as prostate, bladder, lung, breast, colon and non-Hodgkin lymphoma which were the most common malignancies.^4^

Ojha et al. report 69 case of RCC in 34,156 patients diagnosed with MM in Dana-Farber Cancer Institute in Boston during 1973 and 2006. Ojha and his colleagues suggest that MM was 1.51 times more likely to be found in RCC patients than in the general population.^6^

In 2008, Bhandari et al. reported six cases of RCC in their 600 cases of plasma cell dyscrasia during ten years;^4^ Dutcher and Wiernik have reported an increase incidence of hematologic malignancies in families of patients with RCC. Interestingly the large majority (94%) of these hematologic malignancies were B-cell origin.^9^ Our patient’s family history was negative for any malignant disease; although his MM. was diagnosed initially and then RCC. It has been hypothesized that one malignancy produces a tumor stimulating hormone like IL6 or TNFα which may increase the risk of second malignancy.^5^ Padhi and his colleagues have described a 69 year old man

with refractory MM whose serum IL6 was markedly elevated.^10^ Other risk factors may be: environmental factors (tobacco, occupation and pollution, ultra violate light), genetic factors, metabolic syndrome, previous medical treatment, sex and hormonal factors.^11^ Although various hypotheses have been explained the same risk factors as mentioned above, no common etiology has been reported, yet. Besides these, therapeutic strategies employed for MM have been tried for RCC with partial success; which may support the probable common pathophysiology.^4^

In this case report, we have reviewed a patient with concomitant malignancies. It is not obvious which malignancy was first occurred as RCC discovered incidentally in multiple myeloma work up. Because our patient’s RCC was in low stages and operable, we first treated the renal tumor; then, he underwent chemotherapy for his myeloma.

## CONCLUSION

 This finding may be useful for further evaluation of the shared risk factors and etiologies of both malignancies. And hopefully lead to more awareness among physicians that a potential second malignancy in MM may be RCC and vice versa. Therefore, any new lytic bone lesion in a patient with prior renal cell carcinoma should be investigated for potential myeloma; especially when there is not any other metastatic lesion.
